# Physical Therapy Licensure Compact Update

**DOI:** 10.5195/ijt.2019.6298

**Published:** 2019-12-12

**Authors:** Leslie Adrian

**Affiliations:** Federation of State Boards of Physical Therapy, Alexandria, VA, USA

Since the Fall 2017 ITJ article on the Physical Therapy Compact (PTC), much has changed! The PTC came into effect in April 2017 with the tenth jurisdiction passing Compact legislation. Later that year, the Physical Therapy Compact Commission (PTCC) held the first of it's annual in-person meetings. In 2019, the PTC has twenty-six members (www.fsbpt.org/compact) and is fully operational with finalized rules, bylaws, and policies approved by the PTCC to help the PTC run efficiently and effectively.

A hallmark of the PTC is the compact privilege; the authorization to practice or work in the jurisdiction rather than a license. After verifying eligibility, the PTCC issues the compact privilege; the process to apply and receive a Compact Privilege takes minutes. As of December 2019, sixteen jurisdictions are actively issuing and accepting compact privileges (http://ptcompact.org/ptc-states).

